# Characterisation of microbunching instability with 2D Fourier analysis

**DOI:** 10.1038/s41598-020-61764-y

**Published:** 2020-03-19

**Authors:** A. D. Brynes, I. Akkermans, E. Allaria, L. Badano, S. Brussaard, G. De Ninno, D. Gauthier, G. Gaio, L. Giannessi, N. S. Mirian, G. Penco, G. Perosa, P. Rebernik, I. Setija, S. Spampinati, C. Spezzani, M. Trovò, M. Veronese, P. H. Williams, A. Wolski, S. Di Mitri

**Affiliations:** 1 ASTeC, STFC Daresbury Laboratory, Daresbury, Warrington, WA4 4AD Cheshire, United Kingdom; 2 Cockcroft Institute, Sci-Tech Daresbury, Keckwick Lane, Daresbury, Warrington, WA4 4AD United Kingdom; 3 Department of Physics, University of Liverpool, Liverpool, L69 7ZE United Kingdom; 4 ASML Netherlands B.V., De Run 6501, 5504 DR Veldhoven, Netherlands; 5 Elettra-Sincrotrone Trieste S.C.p.A., 34149 Basovizza, Trieste, Italy; 6 Laboratory of Quantum Optics, University of Nova Gorica, 5001 Nova Gorica, Slovenia; 7 LIDYL, CEA, CNRS, Université Paris-Saclay, Saclay, 91191 Gif-sur-Yvette, France; 8 University of Trieste, Dept. Physics, 34127 Trieste, Italy

**Keywords:** Physics, Applied physics, Plasma physics

## Abstract

The optimal performance of high-brightness free-electron lasers (FELs) is limited by the microbunching instability, which can cause variations in both the slice energy spread and longitudinal profile of electron beams. In this paper, we perform 2D Fourier analysis of the full bunch longitudinal phase space, such that modulations in both planes can be studied simultaneously. Unlike the standard 1D analysis, this method is able to reveal modulations in a folded phase space, which would otherwise remain uncovered. Additionally, the plasma oscillation between energy and density modulations is also revealed by this method. The damping of the microbunching instability, through the use of a laser heater, is also analysed with this technique. We confirm a mitigation of the amplitude of modulation and a red-shift of the microbunching frequency as the energy spread added increases. As an outcome of this work, a systematic experimental comparison of the development of the instability in the presence of different compression schemes is here presented for the first time.

## Introduction

The microbunching instability^1–7^ is a collective effect that can develop due to either shot noise or a non-uniform intensity profile of the photo-cathode laser^8–10^ in the injector of high-brightness electron accelerators, such as free-electron lasers (FELs). Small-scale structure that develops in this low-energy regime can then undergo amplification due to longitudinal space-charge (LSC)^11–16^ and coherent synchrotron radiation (CSR) effects^1,2,17–22^, within and following dispersive regions^23^.

This instability is of critical importance for high-brightness electron sources^1,24–26^, as it can both disable diagnostic devices and cause a degradation in the beam quality, which has an adverse effect on the application of the beam. An example of the significance of the energy modulation is in an FEL: if there are discrete energy bands in an electron bunch upon its entrance to the undulator, this may result in the generation of photon pulses that also exhibit these energy bands^27^.

A number of experimental analyses of the instability have been published, using coherent optical transition radiation (COTR)^28–33^, measuring its influence on FEL intensity and gain length^24,34^, and by direct measurements of the longitudinal phase space^3,35–38^. This final method of analysis is particularly useful for benchmarking both simulation codes and analytic models which describe the development of the instability.

Laser heaters have proven to be invaluable components of short wavelength FELs^4,24,34,39^, which are capable of mitigating this instability. The laser heater (LH) in its nominal configuration consists of a small dispersive chicane, in the centre of which is an undulator. Propagating simultaneously with the electron beam in the undulator is a laser pulse which imposes an energy modulation on the beam. Since the wavelength of the laser is much shorter than the electron bunch length, the paths travelled by particles with different energies through the second half of this chicane will then overlap in longitudinal phase space. This process therefore removes the modulation, also causing an effective slice energy spread increase across the bunch, thereby preventing the development of the microbunching instability in the remainder of the accelerator lattice.

In this paper we study the effect of the laser heater in the FERMI FEL^40,41^ for three magnetic bunch compression scenarios, and for a range of laser pulse energies. Since the final bunching amplitude is highly dependent on the accelerator lattice configuration^1^, it is important to consider a range of bunch compression schemes in order to evaluate their feasibility for driving an FEL. Through this experimental analysis of microbunching over a wide range of laser heater and accelerator lattice settings, we can find the optimal working point for suppressing the instability while maintaining a high-quality electron bunch. In order to achieve this, we have developed a novel method of analysing the longitudinal phase space of such beams using two-dimensional Fourier analysis.

The microbunching in the longitudinal phase space of a particle bunch can be described using the so-called bunching factor^1^. When analysing microbunching along the longitudinal axis only, the bunching factor is described by the Fourier transform of the current density of the bunch. In order to extend this analysis to two dimensions, we take the Fourier transform of the longitudinal phase space density, $\rho \left(t,E\right)$:

1 $$b(k,m)=\frac{1}{N}\int \int \rho \left(t,E\right){e}^{-i\left(kt+mE\right)}dtdE,$$

where $N$ is the number of particles, and $k$ and $m$ describe, respectively, the modulation frequency as functions of time and energy.

From this 2D analysis, the plasma oscillation phase of the bunch can be deduced – that is, the interplay between bunching in energy and in time, and how the bunch compression can impact this, can be measured experimentally.

Given an initial microbunching (i.e. variation in density as a function of the longitudinal co-ordinate along the bunch, arising from small variations in the intensity profile of the photoinjector laser, for example) LSC causes electrons to experience a force from high-density regions of the bunch towards low-density regions. As the bunch travels along the beam line, the forces lead to an energy modulation, and the subsequent motion of particles within the bunch (as a result of the variation of velocity with energy) then leads to a reversal between the high-density and low-density regions. The process repeats, with the net result being an oscillation in the beam density (i.e. a plasma oscillation) at the frequency^13,42^:

2 $${\omega }_{P}({k}_{0},\gamma ,{I}_{0},{r}_{b})[{s}^{-1}]=c{\left[\frac{{I}_{0}}{{\gamma }^{3}{I}_{A}}{k}_{0}\frac{4\pi | {Z}_{LSC}({k}_{0},\gamma ,{r}_{b})| }{{Z}_{0}}\right]}^{1/2},$$

with $c$ the speed of light, ${I}_{0}$ the peak current of the bunch, $\gamma$ the Lorentz factor, ${I}_{A}=17045$ A the Alfven current, ${k}_{0}$ the initial modulation wavenumber, ${r}_{b}$ the transverse bunch size, ${Z}_{0}\approx 377$ $\Omega$ the free space impedance, and ${Z}_{LSC}({k}_{0},\gamma ,{r}_{b})$ the LSC impedance. Rotation of the longitudinal phase space in a bunch compressor can lead to microbunching in longitudinal co-ordinate (i.e. at a phase $0$ or $\pi$ in phase space) to become a microbunching in energy (i.e. at a phase $\pi /2$ or $3\pi /2$), or more generally a microbunching in both longitudinal co-ordinate and energy (that is, along an axis at an intermediate angle between $0$ and $\pi /2$ in phase space). The final structure in phase space after bunch compression depends on the initial phase of the plasma oscillation (and energy modulation) and the angle of rotation of phase space in the bunch compressor^28^. As the bunch becomes ultrarelativistic, ${\omega }_{P}$ becomes greatly reduced, meaning that the period of the oscillation between energy and density modulations for a typical linac driver for an FEL can be on the order of $100$s of metres. Nevertheless, the linac length in a machine such as FERMI is comparable to the period of the plasma oscillation. Another factor that can influence the phase of the plasma oscillation is the bunch compression process. As demonstrated below, 2D Fourier analysis of the longitudinal phase space is able to reveal this phase, the control and application of which has applications to both FELs^16,43^ and novel wakefield-based accelerators^44,45^.

## Accelerator and Laser Heater Configurations

**Figure 1:**
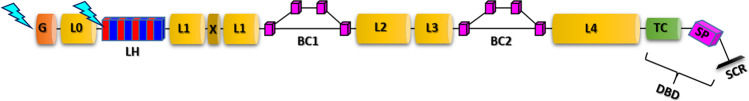
Schematic of the FERMI linac. The beam is produced and accelerated initially in the gun (G), and is subsequently accelerated in linacs L0–4. The laser heater (LH) provides an uncorrelated slice energy spread, from a few keV to tens of keV, between L0 and L1. The two variable bunch compressors are labelled as BC1 and BC2. At the exit of L4, the beam is streaked via the vertical RF deflecting cavity (TC) and observed in the diagnostics beam dump (DBD) line after passing through a horizontal spectrometer dipole (SP) and being imaged on a screen (SCR).

A schematic of the FERMI linac is shown in Fig. 1. Electrons are produced in a high-brightness photo-cathode electron gun, and accelerated in Linac 0 (L0) to around $100$ MeV^46^. The parameters of the laser heater, located at the exit of L0, are given in ref. ^34^. After the laser heater section, the bunch is accelerated in Linac 1 (L1) to an energy of around $300$ MeV – this accelerating section also includes an X-band cavity to manipulate the beam longitudinal phase space, and in turn linearise the compression process. The first bunch compressor with variable ${R}_{56}$, BC1, is located at the exit of this linac, after which point the bunch is further accelerated in the remaining accelerating sections, Linacs 2, 3, and 4^47^. A second variable bunch compressor, BC2, is located between L3 and L4. For the purposes of our experiment, L4 was switched off, meaning that the final beam energy at the diagnostic point was in the range 710–790 MeV.

The beam longitudinal phase space was measured at the Diagnostic Beam Dump (DBD) station (see Fig. 1) by means of a vertical RF deflector (VRFD) located at the end of L4, followed by a horizontal spectrometer magnet (SP)^48^. A screen in the DBD line provides a measurement of the energy of particles in the horizontal plane via the dispersion function of the spectrometer, and the arrival time of the particles in the beam through the calibration of the VRFD.

The temporal and energy resolution at the screen was optimised through careful matching of the beam optics through to the DBD line. In order to calculate the resolution in both planes accurately, errors due to the screen pixel size, the beam non-zero vertical emittance ${\epsilon }_{y}$, and the VRFD-induced energy spread must be taken into account when calculating the slice energy spread (SES). The longitudinal momentum spread induced by the deflector, ${\sigma }_{{\rm{\delta }},VRFD}$, is dependent on the vertical position of particles within the cavity. Given the beam mean energy $E\approx {\bar{p}}_{z}c$, where ${\bar{p}}_{z}$ is the beam mean longitudinal momentum, evaluated at a distance of ${\sigma }_{z}$ from the bunch centroid, the rms value of ${\sigma }_{{\rm{\delta }},VRFD}$ is^49^:

3 $${\sigma }_{\delta ,VRFD}\approx \frac{e{V}_{rf}{k}_{rf}}{2{\bar{p}}_{z}c}\sqrt{\left(\frac{e{V}_{rf}{k}_{rf}}{2{\bar{p}}_{z}c}\right){\frac{L}{3}}^{2}{\sigma }_{z}^{2}+{\epsilon }_{y}{\beta }_{y,VRFD}},$$

with ${V}_{rf}\approx 19$ MV, ${k}_{rf}=62.8$ m${}^{-1}$ the voltage and wavenumber of the VRFD with length $L=3.5$ m, and ${\beta }_{y,VRFD}\approx 25$ m the average vertical betatron function in the VRFD. Measurements of the beam optics parameters, of the SES vs. the RF power attenuation factor of the deflector, and the evaluation of the effective peak deflecting voltage, led to estimated temporal and energy resolutions of $\approx 10$ fs and $70$ keV, respectively.

Three different machine configurations were used for this study, based on which of the variable bunch compressors was used to compress the beam by a total compression factor in the range 32–40: BC1 only, BC2 only, and a combination of BC1 with BC2. For each of these three lattice configurations, the longitudinal phase space was measured for a number of settings of the laser heater power. The lattice and electron beam parameters for all three configurations are provided in Table 1.

**Table 1:** Main lattice and measured beam parameters of the FERMI accelerator at the end of Linac 4 for the three compression schemes.

Bunch parameters	Unit	BC1 only	BC2 only	BC1 + BC2
Bunch charge	pC	$100$	$100$	$100$
Beam energy	MeV	$787$	$713$	$754$
Bunch length (rms)	fs	$54$	$37$	$54$
Chicane bending angle	mrad	$105$	$90$	$105,85$
${R}_{56}$	mm	$-62.5$	$-40.9$	$-62.5,-40.9$
Peak current	A	$560$	$800$	$560$
Relative energy spread (rms)	%	$0.1$	$0.15$	$0.3$
Linear energy chirp at BC entrance	m${}^{-1}$	$\approx -15.5$	$\approx -23.8$	$\approx -11.9,-21.5$
Linear energy chirp at DBD	m${}^{-1}$	$\approx -20$	$\approx -85$	$\approx -110$

## Results and Discussion

### Slice energy spread and laser heater action

**Figure 2:**
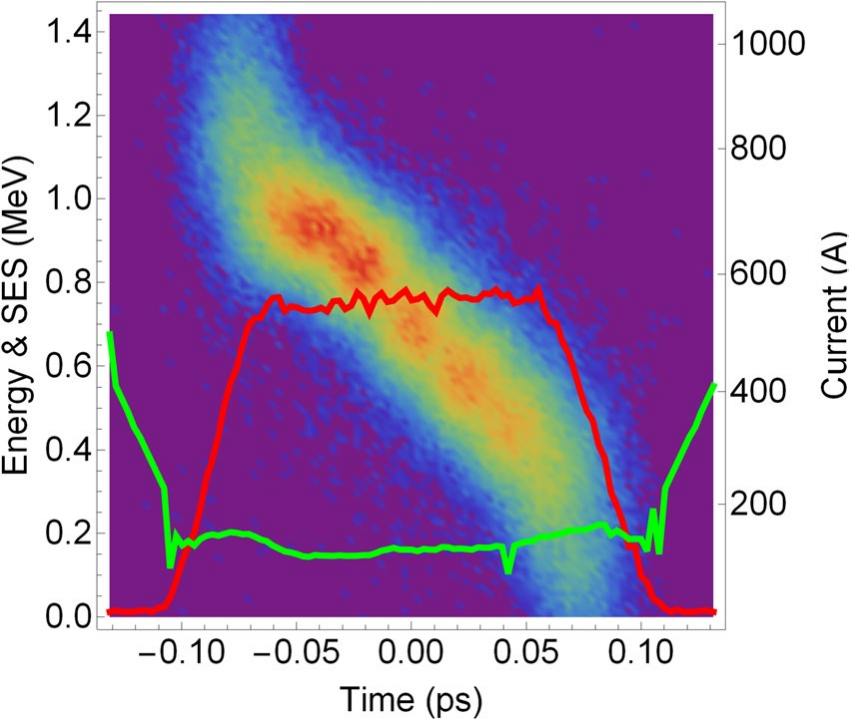
Longitudinal phase space of a typical bunch compressed using BC1 only, with the laser heater switched off. The current profile of the bunch is shown in red, and the slice energy spread is in green.

The influence of the laser heater on the bunch can be quantified both in terms of the SES increase that is imposed on the bunch, and of the effect that this added energy spread has on the microbunching parameters of the beam. These latter parameters include the bunching factor and modulation period, and are discussed in the subsection below. A representative example of a measurement of the SES and current profile of a bunch compressed using BC1 only, with the laser heater switched off, is shown in Fig. 2. Each point of the SES curve represents the rms size of a Gaussian fit to the profile of an image slice, with a width provided by the resolution of the deflector.

**Figure 3:**
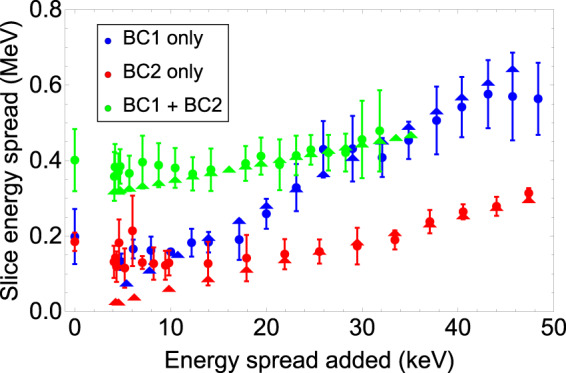
Slice energy spread at the bunch core, measured at the end of the linac, as a function of the energy spread added by the laser heater for all three compression scenarios. Circles show the measured values, and the triangles use Eq. (8) of Ref. ^13^, taking into account the decompression by the spectrometer dipole.

The SES at the DBD screen as a function of the laser heater energy added is shown in Fig. 3 for all three compression scenarios. In this case, only the mean SES of the bunch core has been calculated, since this parameter is sometimes observed to increase by a large amount at the head and tail of the bunch due to strong nonlinear compression, typically associated with the generation of current spikes.

First, we notice that the SES associated with null or weak LH action (up to $10$ keV of added energy spread) is comparable in the BC1 and BC2 schemes, and much larger in the BC1 + BC2 scenario, in spite of the lower total compression factor compared to the single stage compressions. This is consistent with theoretical predictions of the microbunching gain, according to which, once the peak current is partially increased by the first compressor, the second compressor then causes the energy modulation cumulated upstream to be converted into amplified bunching^1,17^. This in turn drives larger energy modulations, resulting in a larger SES at the linac end. Given that the CSR-induced emittance growth is not expected to be significantly larger for the double compression scheme, the larger SES in this case is an indication of stronger instability gain.

At the same time, in spite of a higher final peak current in the BC2-only scheme with respect to BC1-only, the SES (for null or weak LH power) is comparable in these two cases. This is due to a counterbalancing of the higher instability gain due to a larger final peak current for the BC2-only scheme with the lower instability gain due to the bunch propagating with a much lower peak current (around $18$ A) up to the entrance of BC2. In the case of BC1-only, the bunch is compressed earlier, and therefore the short bunch travels for a longer distance, but with a lower peak current, and thus the gain is comparable in both cases. This allows one to conclude that the final peak current level is not the only ingredient for inferring a higher instability gain. In addition, the evolution of the beam properties along the entire beam line must be considered.

Second, we note that at very strong heating (with larger than $20$ keV added energy spread) the instability is expected to be partly or fully suppressed, and therefore the SES is expected to follow a linear dependence from the LH-induced energy spread, where the gradient should be proportional to the linear compression factor (this is a consequence of the approximate preservation of the beam longitudinal emittance^50^). Though such dependence is apparent in the figure, the slope is not as steep as expected. The reason for this is that the bunch length increases as the beam passes through the DBD spectrometer magnet, in the presence of a relatively large linear energy chirp (see Table 1). In this case, the bunch length can be reconstructed from the beam image at the screen as it was at the deflector location (i.e. at the nominal compression factor).

The SES, instead, is decreased by the same factor by which the bunch is lengthened in the dipole magnet. The analysis of the images for the three compression schemes confirms that the energy chirp $h=\frac{dE}{Edz}$ (for a beam energy $E$) at the dipole is approximately $20$ m${}^{-1}$, $85$ m${}^{-1}$ and $110$ m${}^{-1}$ for the BC1, BC2 and BC1 + BC2 scheme, respectively. Once coupled to the dipole longitudinal dispersion, ${R}_{56}=0.12$ m, that chirp reduces the nominal compression factor, and therefore the SES cumulated up to that point, by factors of approximately $3$, $13$ and $16$ respectively. This scaling only holds in the region of strong beam heating, which allows the longitudinal emittance to be approximately preserved during magnetic (de-)compression. When taking this decompression into account, we find good agreement between the measured values and theoretical calculations for the LH-induced energy spread increase using Eq. (8) of Ref. ^13^. For heating levels lower than $20$ keV, the microbunching instability is still playing a role. In fact, a minimum of the SES is just visible for the single compression schemes, in spite of the relatively large error bars, for the LH set at around 5–8 keV.

**Figure 4:**
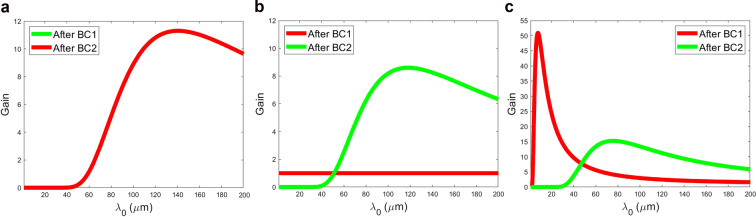
Calculated microbunching gain as a function of initial modulation wavelength ${\lambda }_{0}$ for the three compression schemes, including the effects of LSC^13^, CSR^1,17^ and IBS^53^ (**a**) BC1 only. (**b**) BC2 only. (**c**) BC1 + BC2.

Theoretical evaluations of the gain curve associated with the CSR impedance only as introduced in^1,17^, indicate that, for all the three compression schemes, the CSR-induced microbunching is negligible compared to the effect of the LSC impedance^13^: the peak CSR gain is typically around unity, and is one or two orders of magnitude lower than that associated with LSC. Consequently, the impact of CSR on the final SES is also very small (for an estimate of the SES and its dependence on the total gain, see e.g., Eq. (17) in Ref. ^3^). The gain in microbunching as a function of initial modulation wavelength is plotted for the three compression schemes in Fig. 4, taking into account the beam and lattice parameters through the machine. The calculation takes into account CSR and LSC effects, and additionally the effect of intrabeam scattering (IBS)^51,52^ – a full study of this final effect is underway^53^. It can be seen that maximum gain for the single compression schemes are around half that of the double compression scheme.

Particle tracking runs for the FERMI injector have been conducted with the General Particle Tracer (GPT) code^54^ (for more details on these simulations, see Ref. ^23^). These simulations provide an estimate of $2$ keV in the bunch core for the uncorrelated (slice) beam energy spread out of the injector. In the presence of a total compression factor of $35$ as in the BC1-only case, for example, and in the absence of the instability, the preservation of the beam longitudinal emittance^50^ predicts a final value around $70$ keV. The measured minimum SES is larger than this, as a signature of residual instability action in the longitudinal phase space at low heating levels. The error bars are dominated by the uncertainty on the measurement reproducibility.

### Fourier analysis of longitudinal phase space

Two-dimensional Fourier analysis of the longitudinal phase space of a beam can reveal, in addition to the microbunching frequency and amplitude, the phase of the plasma oscillation between bunching in energy and in time. By comparing these parameters for measured bunches, it is possible to demonstrate experimentally the influence of collective effects on the microbunching structure in these bunches, and to address the accuracy of the models used in simulation.

**Figure 5:**
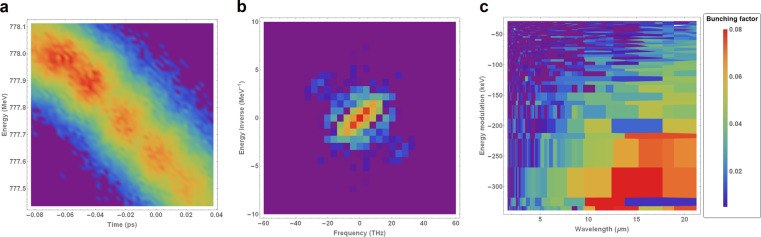
Example of 2D microbunching analysis for a bunch compressed using BC1 only, with the laser heater off. The two satellites located around $\left[\pm 20\ {\rm{THz}},\mp 3.5\ {{\rm{MeV}}}^{-1}\right]$ in the middle plot represent the modulations in intensity that are visible in a. (**a**) Longitudinal phase space. (**b**) Fourier spectrum of a in frequency space. (**c**) Zoomed-in Fourier spectrum of a in wavelength space - mean of 20 shots. (**a**) Longitudinal phase space. (**b**) Fourier spectrum of  (a) in frequency space. (**c**) Zoomed-in Fourier spectrum of (a) in wavelength space (mean of 20 shots).

The microbunching parameters of interest can be extracted using the following procedure: (1) zoom in on the beam image to remove low-frequency components on the order of the bulk scale of the bunch, and apply an intensity threshold to suppress noise; (2) apply a 2D Fourier transform to this image and convert from frequency to wavelength, and from dimensions of inverse energy space to dimensions of energy (via the reciprocal of the energy inverse axis in Fourier space); (3) select a region of interest based on the position of the satellites around the central term in frequency space; (4) remove wavelength/energy modulation values corresponding to frequencies smaller than half of the Fourier transform of the bunch length and energy spread; (5) find the maximum bunching factor as a function of wavelength/energy modulation. The fourth step in this procedure is necessary to ensure that only truly periodic features contribute to the microbunching analysis. Artefacts associated with noise in the imaging system cannot be removed completely, but a low-intensity threshold is sufficient to remove some of the persistent noise in the Fourier transform. An example to illustrate the application of the procedure outlined above to a beam phase space image – compressed using BC1-only – is shown in Fig. 5.

The normalised amplitude in Fourier space yields the bunching factor and the distance from the central term gives the periodicity along both axes. The arctangent of the ratio between these values represents the plasma oscillation phase – we normalise the units in this case such that the phase is dimensionless (see below). The physical origin of this parameter is a combination of a periodic oscillation between energy and density modulations and the shearing of microbunches due to the magnetic bunch compression process, as mentioned above. Therefore, the term ‘phase’ may not be strictly applicable. Nevertheless, since these two effects combine to produce a periodic variation in the longitudinal phase space with only a single step change due to bunch compression, we reserve the term ‘phase’ to refer to the tile angle of the separate microbunches in longitudinal phase space.

An analytic model of a modulated Gaussian bunch has been constructed for the purposes of demonstrating the influence of these beam parameters on the Fourier transform – see the Appendix for examples. Both low- and high-frequency peaks in intensity are of less interest, since the former relate to structure on the scale of the bunch (including current spikes at the head and tail of the bunch), and the latter arise primarily due to the resolution of the imaging system.

The Fourier transform of the original image of the longitudinal phase space of the bunch in Fig. 5a– in this case, compressed using BC1 only, and with the laser heater switched off – is shown in Fig. 5b. In this case, the modulation on the bunch is represented by the points at $\approx [\pm 20{\rm{T}}{\rm{H}}{\rm{z}},\mp 3.5{{\rm{M}}{\rm{e}}{\rm{V}}}^{-1}]$ in Fig. 5b– this can be confirmed by applying a low-intensity threshold to Fig. 5b and performing an inverse Fourier transform, in which case only the DC term in the Fourier transform remains, and the modulations on the bunch disappear. The symmetric properties of Fourier analysis mean that it is arbitrary which of the two satellites in Fourier space we choose to analyse.

By transforming to wavelength/energy space (taking the reciprocals of the values along the axes in Fig. 5b into wavelength/energy space), averaging over $20$ shots, and zooming in on the region of interest, the microbunching features in both dimensions can be clearly seen – see Fig. 5c. Since the dimensions of Fig. 5c are the reciprocal dimensions of the initial Fourier transform, the pixels in this plane become larger as the wavelength and energy values increase. The red region in Fig. 5c represent the strongest density modulations in the longitudinal phase space of the bunch.

**Figure 6:**
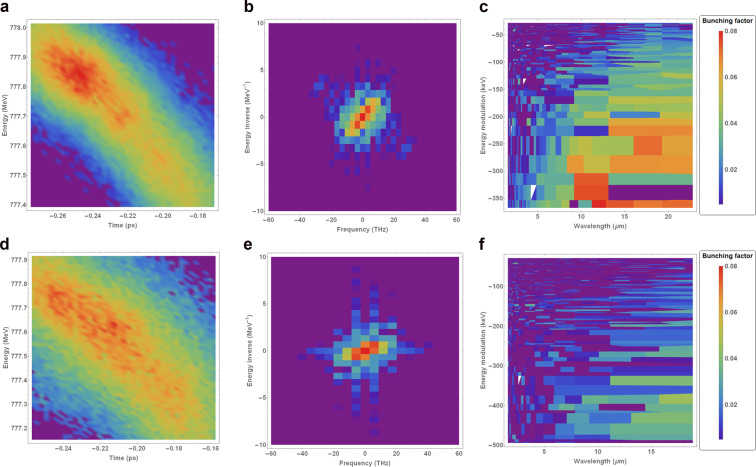
Examples of 2D microbunching analysis for a bunch compressed using BC1 only, with the laser heater on, adding: Top row: $5$ keV; Bottom row: $26$ keV. The order of the plots from left to right is the same as that of Fig. 5. (**a**) Longitudinal phase space. (**b**) Fourier spectrum. (**c**) Zoomed-in Fourier spectrum in wavelength-energy modulation space. (**d**) Longitudinal phase space. (**e**) Fourier spectrum. (**f**) Zoomed-in Fourier spectrum in wavelength-energy modulation space.

As the power of the laser heater is increased, the uncorrelated energy spread of the bunch increases. This results in a reduction in intensity of the satellites in the Fourier space that represent the density modulations on the bunch. Examples of images of beams that have been heated are shown in Fig. 6 – these measurements were also made with BC1-only compression. It can be seen that, even with a small added energy spread of $5$ keV, the microbunching has been suppressed by around a factor of $2$, while for a much larger laser heater power of $26$ keV, the slice energy spread of the bunch increased by a large amount, and the microbunching level has essentially been suppressed to the noise level. The colour scale on Figs. 5c and 6c,f is the same, in order to enhance the visibility of the suppression of density modulations. Due to noise in the imaging system, the measured bunching of a background image still exhibits a maximum measured bunching of around 0.01–0.02 (see the colour scale on Fig. 6f), and so this can be said to demonstrate cases where there is no measurable microbunching in a real beam image.

### Spectral bunching and plasma oscillation phase

**Figure 7:**
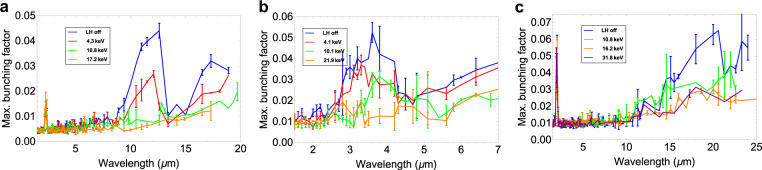
Maximum measured bunching factor in the wavelength axis for bunches compressed using all three machine configurations, for a number of laser heater energy settings (average of 20 shots). (**a**) BC1 only. (**b**) BC2 only. (**c**) BC1 + BC2.

In order to provide a comparison of the microbunching structure for different settings of the laser heater pulse energy, we project the 2D bunching factor onto the wavelength axis. These projections are shown in Fig. 7 for all three compression schemes. Due to the inherent noise associated with microbunching, each point in the plots represents the mean microbunching over $20$ shots as a function of the compressed wavelength. The trend towards decreasing bunching as the laser heater energy increases is clear, and it approaches the noise floor of the measurement for a relatively small added energy spread. We can also compare the final modulation wavelength with the theoretical gain curves shown above (Fig. 4). Given the compression factor of around $37$ for the BC2-only case, we see good agreement between the wavelength at the peak bunching factor, whereas the agreement is not as close for the BC1-only case, which had a slightly smaller compression factor of around $32$.

We also note that the characteristic frequency of the microbunching is a 2-dimensional parameter, being defined both by the phase of the plasma oscillation and the radius of the circle that defines the position of the microbunching satellite in Fourier space. As such, for cases with an intermediate bunching between energy and density modulations, simply projecting the bunching along one axis will present a distorted picture of the final microbunching period. This is most evident in the discrepancy between theoretical and measured values of the peak bunching wavelength for the double compression scheme (Fig. 7c). Nevertheless, such projections can provide a useful illustration of the action of the laser heater.

By analysing the behaviour of the bunching projected onto the wavelength axis, we observe that the maximum bunching values for the two single-compression schemes are comparable. In the BC1 + BC2 compression option, the largest bunching is observed, and the largest energy spread over all three cases must be added to reduce the bunching to the measurement noise level.

**Figure 8:**
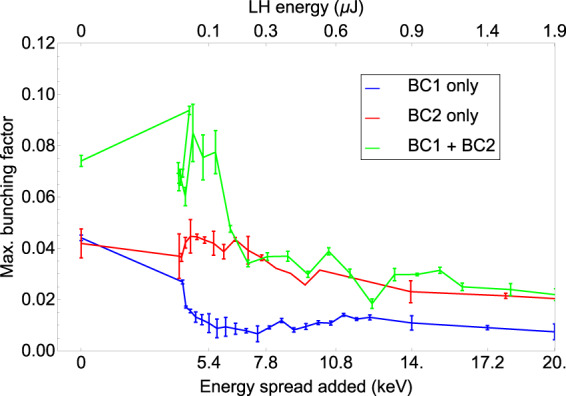
Maximum bunching factor as a function of laser-heater induced energy spread for all three compression scenarios.

If the maximal bunching factor is then calculated as a function of laser heater pulse energy (as shown in Fig. 8), it can clearly be seen that the microbunching has been suppressed for a relatively low added energy spread – around 10–14 keV – for all three compression scenarios. It is also noted that, in accordance with the theoretical calculations in Fig. 4, the maximum bunching factor with the laser heater off is largest for the double compression scheme, almost a factor of two larger than the single-compression cases. On comparing this increase in energy spread added with the results shown in Fig. 3, it can be seen that this small amount of beam heating is sufficient to remove the microbunching structure in the beam without increasing the slice energy spread of the bunch by a large amount. For a compression factor of 35, an added energy spread of $10$ keV means increasing the overall energy spread of the bunch by around $0.5$ %.

The position of maximal bunching factor in Fourier space can also be used to deduce the plasma oscillation phase of the modulations on the beam. As mentioned above, the oscillation between microbunching in energy and density, and the bunch compression process, can cause a shearing of the microbunches in longitudinal phase space. This manifests in the Fourier transform of the beam image as a rotation of the microbunching satellites in Fourier space around the central term.

**Figure 9:**
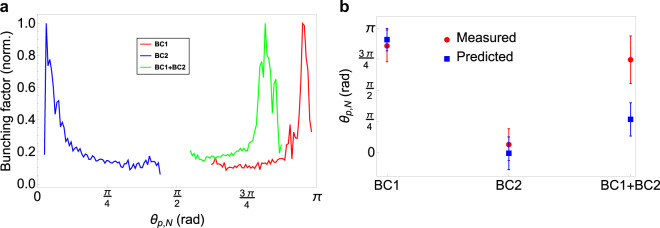
Bunching factor as a function of normalised plasma oscillation phase ${\theta }_{P,N}$ and a comparison between measured and predicted final plasma oscillation phase for all three compression scenarios. (**a**) Normalised plasma oscillation phase - average of 20 shots. (**b**) Plasma oscillation phase at the DBD location - measured (red circles) and theoretical (blue squares).

Since the modulation amplitude in energy is on the order of $100$s of keV, while the modulation period of the peak bunching factor is on the order of $10$s of ${\rm{\mu }}$m, we have measured the ‘normalised’ plasma oscillation phase as ${\theta }_{P,N}={\tan }^{-1}({\bar{E}}_{mod}^{i}/{\bar{f}}_{mod})$, where ${\bar{E}}_{mod}^{i},{\bar{f}}_{mod}$ denote the normalised modulation along the energy-inverse and frequency axes in Fourier space, respectively. The values were normalised with respect to the bunch length and energy spread of the beam, using a method analogous to that used in transverse phase space tomography analysis^55^. This normalised phase for the maximum bunching factor across the three compression schemes (with the laser heater off) is shown in Fig. 9a. In all cases, there is some mixing between microbunching in longitudinal density and in energy. Little variation in the plasma phase was observed when increasing the energy spread imposed by the laser heater.

In order to compare the measured plasma oscillation phase at the DBD location with theoretical predictions, a semi-analytic model has been developed to track the evolution of the plasma oscillation through the machine. Particle tracking runs of the post-injector lattices for the three compression schemes have been conducted using the ELEGANT code^56^ to compute the beam size, peak current and beam energy throughout the lattice. These parameters can be fed into Eq. (2) to calculate, piece-wise, the evolution of the plasma period $c/{\omega }_{P}$ throughout the machine as a function of initial modulation wavelength ${\lambda }_{0}$, also taking into account the shearing of the microbunches due to the bunch compression process as described above. Since any initial modulations on the bunch arising due to shot noise in the injector will be broad-band, we suppose that the normalised plasma oscillation phase ${\theta }_{P,N}$ at the injector exit is zero for all ${\lambda }_{0}$. A comparison between the predicted and measured values for the plasma oscillation phase at the DBD location is shown in Fig. 9b. The error bars on the measured values represent the FWHM of the curves in Fig. 9a, and the errors in the predictions represent the variation in ${\theta }_{P,N}$ across a range of ${\lambda }_{0}$ given by the theoretical curves in Fig. 4. It can be seen that the predictions match up well with the measured values in both single compression schemes, whereas there is a larger discrepancy in the case of double compression. A full investigation of the origins of these plasma oscillation phases will be the topic of a future study.

## Conclusion

In this paper, a novel method of analysing microbunching in the longitudinal phase space of an electron bunch has been developed and applied to experimental data collected at the end of the FERMI linac. Through the use of 2D Fourier analysis, we have extracted the modulation parameters in both time and energy. This technique has provided a method for determining the phase of the plasma oscillation between energy and density modulations for the first time. We have also provided a systematic comparison of the effect of the bunch compression scheme on the microbunching measured at the end of the linac, showing that, in the range of parameters adopted at FERMI, the use of multiple bunch compressors results in a stronger microbunching amplitude than single-compression schemes.

It is expected that this full analysis of the longitudinal phase space of microbunched beams will lead to a deeper understanding of both the conditions for the development and propagation of the microbunching instability, and of the effect that the instability can have on the photon pulses produced in an FEL^27,57^. This could potentially lead to further developments in both the mitigation and exploitation of the instability, further enhancing the capabilities of high-brightness electron accelerators.

**Figure 10:**
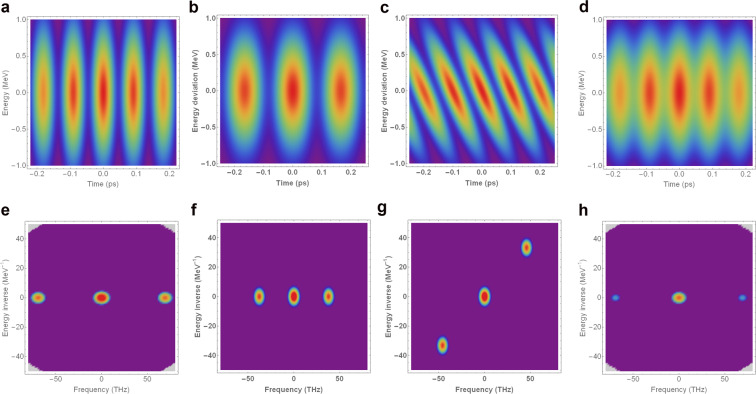
Top row: Model longitudinal phase spaces of a microbunched beam with varying microbunching parameters. Bottom row: Fourier representation of these phase spaces. (**a**) Initial phase space. (**b**) Decrease in modulation frequency *ω*. (**c**) Variation of plasma oscillation phase *θ*. (**d**) Decrease in bunching factor *b*. (**e**) Fourier transform of a. (**f**) Fourier transformof b. (**g**) Fourier transform of c. (**h**) Fourier transform of d.

**Figure 11:**
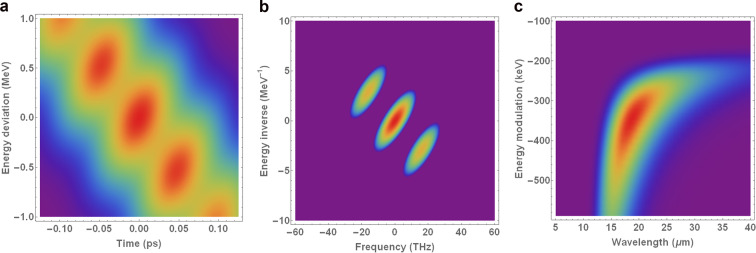
Simulated reproduction of the microbunching analysis shown in Fig. 5. (**a**) Longitudinal phase space. (**b**) Fourier transform. (**c**) Conversion of units of b, with a focus on one of the satellites in Fourier space.

## Appendix: Analytic Model of a Microbunched Beam

A deeper understanding of the Fourier representation of the electron beam longitudinal phase space can be gained by first considering a simple analytic model of the phase space. We begin by considering a 2D Gaussian phase space, modulated by a sinusoidal function. In our model, four parameters can be varied independently to recreate phase space distributions similar to those in a real image:

- Modulation frequency $$\omega $$.
- Linear energy chirp of the bunch $h$.
- Plasma oscillation phase of the microbunches $\theta$.
- Intensity profile of the microbunches, represented by the bunching factor $b$.

We define a bunch with a chirp $h$, a plasma oscillation phase $\theta$, a modulation frequency $\omega$, a bunching factor $b$, and using the following terms for a particle $i$ in the bunch with size ${\sigma }_{t}$ and ${\sigma }_{\delta }={\sigma }_{E}/{E}_{0}$ in the time and energy dimensions, respectively:

4 $$\begin{array}{lll}{t}_{i}(t,\delta ,h) & = & (\bar{t}-t)\cos (2\pi h)+(\bar{\delta }-\delta )\sin (2\pi h),\\ {\delta }_{i}(t,\delta ,h) & = & -(\bar{t}-t)\sin (2\pi h)+(\bar{\delta }-\delta )\cos (2\pi h),\end{array}$$

with $\bar{t}$, $\bar{\delta }$ the central time and energy values. We then arrive at the following definition of a sinusoidally modulated two-dimensional Gaussian function ${\bf{X}}$, which represents the bunch longitudinal phase space:

5 $${\bf{X}}(t,\delta ,h,\theta ,{\sigma }_{t},{\sigma }_{\delta },b,\omega )=\exp \left[-\left(\frac{{t}_{i}{(t,\delta ,h)}^{2}}{2{\sigma }_{t}^{2}}+\frac{{\delta }_{i}{(t,\delta ,h)}^{2}}{2{\sigma }_{\delta }^{2}}\right)\right]\left(1+b\cos {[2\pi \omega {t}_{i}(t,\delta ,\theta )]}^{2}\right).$$

The Fourier transform of Eq. (5) can then be used to observe the effect of varying the microbunching parameters in order to better understand the phase spaces of real beams. The top row of plots in Fig. 10 show each parameter being varied independently of the others, save for the chirp parameter $h$ – this is of less interest for analysing the structures within the bunch. A comparison of each set of plots, and their respective Fourier transforms, will help in understanding the properties of measured longitudinal phase spaces. It can be seen from these four sets of images that it is possible to isolate different properties of the bunch from their Fourier transforms. In every image, the DC term is given by a bright spot in the centre of the Fourier space. This would be present even in the Fourier transform of a purely random distribution of pixels. The microbunching structure is evident in the sidebands of the Fourier transform.

As seen in Fig. 10a,b as the modulation frequency increases, there are a greater number of microbunches present along the bunch in real space, and in the Fourier representation, the sidebands become increasingly separated from the central spot (compare Fig. 10e with Fig. 10f). This means that the short-wavelength terms will be further separated from the DC term than long-wavelength terms. If the microbunches themselves are rotated while the bulk rotation (or energy chirp) is kept constant, as in Fig. 10c, the Fourier sidebands themselves rotate around the DC term while their orientation remains constant (see Fig. 10g). This demonstrates how the plasma oscillation phase can be measured. Decreasing the modulation amplitude (which is analogous to the bunching factor) causes the microbunches to become less discrete within the bunch, as shown in Fig. 10d. In Fourier space, the sidebands become less bright (as shown in Fig. 10h). Finally, it shown in Fig. 11 that this simplified analysis technique can yield results analogous to those for real bunches, with this example reproducing the structure within the longitudinal phase space given in Fig. 5a, and its associated Fourier transform (in Fig. 5b,c).

This simple model has demonstrated that the Fourier representation of the phase space of an electron bunch can reveal the properties of the microbunching structure. The model does not take into account any nonlinearities in the longitudinal phase space that arise due to imposing an energy chirp onto the bunch; however, such features do not greatly distort the Fourier representation of the longitudinal phase space.
